# SANS: high-throughput retrieval of protein sequences allowing 50% mismatches

**DOI:** 10.1093/bioinformatics/bts417

**Published:** 2012-09-03

**Authors:** J. Patrik Koskinen, Liisa Holm

**Affiliations:** ^1^Department of Biosciences, Division of Genetics; ^2^Institute of Biotechnology, University of Helsinki, 00014 Helsinki, Finland

## Abstract

**Motivation:** The genomic era in molecular biology has brought on a rapidly widening gap between the amount of sequence data and first-hand experimental characterization of proteins. Fortunately, the theory of evolution provides a simple solution: functional and structural information can be transferred between homologous proteins. Sequence similarity searching followed by *k*-nearest neighbor classification is the most widely used tool to predict the function or structure of anonymous gene products that come out of genome sequencing projects.

**Results:** We present a novel word filter, suffix array neighborhood search (SANS), to identify protein sequence similarities in the range of 50–100% identity with sensitivity comparable to BLAST and 10 times the speed of USEARCH. In contrast to these previous approaches, the complexity of the search is proportional only to the length of the query sequence and independent of database size, enabling fast searching and functional annotation into the future despite rapidly expanding databases.

**Availability and implementation:** The software is freely available to non-commercial users from our website http://ekhidna.biocenter.helsinki.fi/downloads/sans.

**Contact:** liisa.holm@helsinki.fi.

## 1 INTRODUCTION

*k*-nearest neighbor classifiers are used widely and successfully to infer the function of newly sequenced proteins. Neighbors are commonly determined by sequence comparison. Protein sequence databases have grown so large that retrieving the neighbors of a query sequence is prohibitive in the exact mode and takes 1–2 min using popular heuristics [BLAST (Altschul *et al.*, 1990)]. With several thousand proteins in each new genome, ‘blasting’ consumes considerable resources in contemporary bioinformatics research. Strategies for database searching fall into five categories:

1. All versus all pairwise comparisons using exact alignment [e.g. SSEARCH (Pearson, 1991)] or generating fast approximate alignments [e.g. FASTA (Pearson, 1991)].
2. Comparison against a representative subset rather than all proteins (e.g. Holm and Sander, 1998; Li and Godzik, 2006; Park *et al.*, 2000).
3. Comparison against a library of profile models of protein families (e.g. Punta *et al.*, 2012; Quevillon *et al.*, 2005).
4. Using word filters to eliminate dissimilar sequences from comparison by exact alignment (e.g. BLAST).
5. Ranking database sequences using a simple feature vector distance [e.g. USEARCH (Edgar, 2010); this work].

**Fig. 1.**
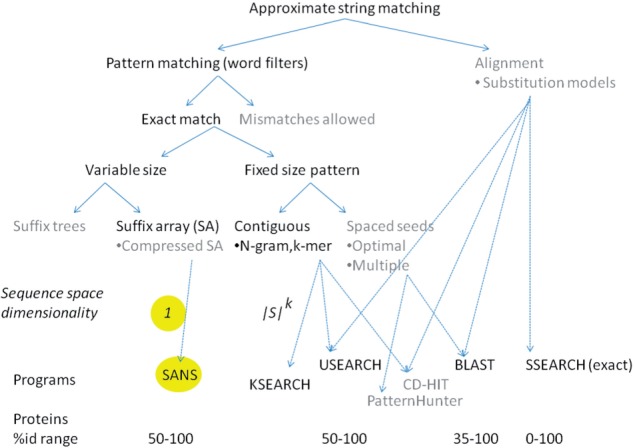
A classification of approaches used by representative application programs for protein sequence retrieval. This work focuses on word filters based on suffix arrays or *k*-mer counting. Related approaches or data structures are shown in gray and not expanded. SANS is simple and fast but has a limited application range. Other fast programs calculate explicit alignments for a filtered subset of database proteins. SSEARCH calculates the optimal alignment between the query and all database proteins

The speed of the methods increases toward the bottom of the list. USEARCH is orders of magnitude faster than BLAST, and we report here a novel method which is 10 times faster than USEARCH. The fast methods are based on word filters. Conserved amino acids are often ‘clumped’ in homologous protein sequences so that there is a good probability of finding identical *k*-mers (Table 1). Many practical tools combine word filters with explicit alignment of a filtered subset of the database (Fig. 1).

**Table 1.** Longest run of identically aligned amino acids in optimal alignment

Pairwise identity (%)	95th percentile	90th percentile	50th percentile	Number of pairs
<30	2	2	3	23 383 124
30–50	2	3	5	15 795 276
50–70	5	6	12	1 405 454
70–90	12	14	26	633 947
>90	19	27	58	88 588

Pairs with *e*-value <1 from the genome benchmark dataset were categorized based on the sequence identity in optimal pairwise alignment by SSEARCH. Longer word lengths increase the selectivity of a filter at the cost of a drop in sensitivity. For example, 12-mers would detect 95% of all pairs with 70–90% overall sequence identity but only half of those with 50–70% identity.

Very short words obviously occur by chance in unrelated sequences. Unique occurrences are expected when 20^k^ is greater than the size of the database, *k* being the length of the word. On the other hand, the sensitivity of a word filter decreases with longer words. Spaced (k,l)-seeds are a special class of word filters with length l and the selectivity of a *k*-mer (Ilie and Ilie, 2007; Ma *et al.*, 2002; Mak and Benson, 2009).

We show experimentally that *k* = 6 is the sweet spot with respect to the current Uniprot database and that sensitivity/selectivity drop either side of this value. The choice of *k* is thus critical for the performance of a word filter. In this work, we test a novel idea which dynamically adjusts word size. It is based on suffixes (substrings starting at position I in a sequence and continuing to the terminator). A suffix array is a data structure which orders the suffixes of a text (protein sequence database) in lexicographic order. Neighboring suffixes share the longest common prefixes. The suffix array is a one-dimensional representation of sequence space. Our idea is to select a window around a query suffix from the suffix array to identify neighbors. The suffix array neighborhood has a constant size. In contrast, neighborhoods defined in terms of *k*-mer word vectors grow larger as the database grows larger.

Suffix arrays are used a lot in the analysis of nucleotide sequences (short read mapping, contig assembly, genome alignment, EST clustering) (e.g. Burkhard *et al.*, 1999). The analysis of protein sequences differs from that of nucleotide sequences in that the range of interesting similarities extends to much higher levels of mismatches. Suffix trees/arrays and related indices have been used previously to organize (Gonnet *et al.*, 1992), search (Califano and Rigoutsos, 1993) and align (Bejerano and Yona, 1999) protein sequences, but they have not supplanted popular tools like BLAST.

**Fig. 2.**
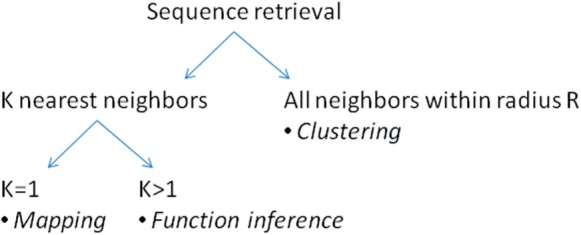
Problem formulations with typical application domains in *italics*

This article is organized as follows. We present comprehensive benchmark tests using real-world data from metagenome samples (6 million proteins), a bacterial genome (4000 proteins) and the uniprot database (18 million proteins). We compare BLAST, USEARCH and many variants of word filters. We characterize the *feasible regime* for word filters and report the fastest known mapping tool for proteins. Finally, we discuss potential applications in function inference (Fig. 2).

## 2 SYSTEM AND METHODS

### 2.1 Protein datasets

We selected real datasets to get a realistic distribution of protein lengths, composition and protein family sizes (Table 2).

**Table 2.** Protein datasets

Name	Proteins	Total length	Average length	Maximum length
Genome	4173	1 405 725	337	6078
Swissprot	533 049	189 597 274	356	35 213
Metagenome	6 050 065	1 219 427 250	202	7557
Uniprot	18 748 263	6 165 066 274	329	36 805

Uniprot is the major collection of protein sequences. It consists of two parts, swissprot and trembl. Swissprot contains manually curated, well annotated sequences. Trembl contains protein sequences that are translated from nucleotide sequences and automatically annotated.

The metagenome dataset is a collection of proteins detected in environmental samples and was downloaded from NCBI (env_nr). Metagenomic sequences typically come from uncultured organisms that are not present in the protein databases.

The genome dataset contains the proteins predicted in a newly sequenced bacterium of the family *Dickeya*. It was chosen as a query set because it belongs to a small clade of *Dickeya* and *Pectobateria*. Although thousands of bacterial genomes have been sequenced, other bacteria typically have ~50% or lower sequence identity to *Dickeya*. Thus, we get a representative distribution of sequence pairs at different ranges of sequence identity (Table 1).

### 2.2 Evaluation

The benchmarks test the ability of tested methods to retrieve acceptable hits to proteins in the query set from the protein database. Our methods are designed to output the top-H hits. The metagenome benchmark evaluates the best hit (*H* = 1) and the genome benchmark evaluates 1000 best hits (*H* = 1000).

We used SSEARCH to compare ‘genome versus uniprot’ and generate a reference of truth for the genome benchmark. True (T) hits are those pairs that have an *e*-value *<*1 by SSEARCH. We assume that method X returns a ranked list of hits (ordered by the method's native score, best hits at the top). TP is the number of true hits in the top-P hits by method X. We define precision = TP/P and recall = TP/T. Both precision and recall range from 0 to 1. Plotting precision against recall yields a curve. The area under that curve is AUC and its maximum value is 1. We compute AUC separately for each query protein and use the average to compare the performance of different methods.

The benchmark set has 41 million TRUE pairs (*e*-value *<* 1) of which 3.6 million rank in the top-1000 by SSEARCH. Different methods need not report identical sets of top-1000 hits. The largest family is ABC transporters (59 988 hits to one query) and the average number of hits per query is 9991.

The metagenome benchmark compares ‘metagenome versus swissprot’. We used BLAST to compute a reference of truth for the metagenome benchmark. Comparison of BLAST to SSEARCH on the genome benchmark showed that BLAST gives a very good approximation of SSEARCH. The whole metagenome dataset consists of 6 million queries. Of these, 3.6 million queries belong to small families for which BLAST reported the complete set of TRUE hits (*e*-value *<* 1). Small families have less than 250 members. The evaluation tests whether the top hit reported by method X belongs to the TRUE set.

### 2.3 Database search programs

We compare three published programs to two methods implemented in this work (cf. Fig. 1). SSEARCH computes the optimal alignment between every sequence in the query set and every sequence in the database. BLAST uses a sophisticated set of word filters to eliminate dissimilar sequences from comparison and computes the optimal alignment. The results of SSEARCH and BLAST are sorted on *e*-value. USEARCH (Edgar, 2010) sorts database sequences by the number of 5-mers they share with the query sequence and tests a few top hits. The search terminates after *α* accepts (*e*-value ⩽ 1) or *r* rejects (*e*-value *>* 1). We used the program with the default values *α* =1, *r* = 8. Section 3.2 describes suffix array neighborhood search (SANS) and KSEARCH which we implemented in-house.

## 3 ALGORITHM

### 3.1 Database indexing

**Fig. 3.**
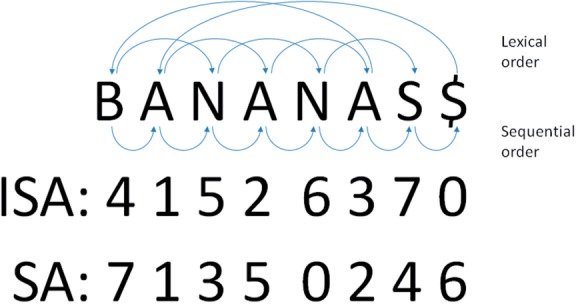
Schematic illustration of suffix array (SA) and inverse suffix array (ISA) on text ‘BANANAS$’. For example, the suffixes ‘ANANAS$’, ‘ANAS$’ and ‘AS$’ are adjacent in the lexically ordered suffix array

A suffix array is an array of integers giving the starting positions of suffixes of a text in lexicographic order. The suffix array SA and inverse suffix array ISA enable jumping between the lexicographic order (*l*) and sequential order (*s*) as follows: SA[*l*] = *s* and ISA[*s*] = *l* (Fig. 3). In this work, the text TXT is a concatenation of protein sequences in alphabet S. The implementation uses FASTA files as input. These are stripped of annotation to create sequence text files (TXT, TXT_Q_) and protein pointers (START, START_Q_). Subscript Q refers to the query set, the database is without subscript. Using Python-like syntax, the sequence of the *i*^th^ protein is the substring TXT[START[*i*]:START[*I* + 1]]. Suffix arrays (SA, SA_Q_) are created on the texts using the recursive sais-lite algorithm (Nong *et al.* 2009). The inverse index ISA is generated by a linear scan of SA using the relation ISA[SA[*l*]] = *l.* We generate an auxiliary index SAP that holds the protein labels of suffixes in lexicographic order. SAP is generated using the relation SAP[ISA[*s*]] = xprot where START[xprot] *< s* ⩽ START[xprot + 1]. In order to compare TXT_Q_ to TXT, we determine the places in SA where suffixes from TXT_Q_ should be inserted. Let *q* be a query suffix starting at position *s*_Q_ in TXT_Q_. We find ISA_mapped_[*s_Q_*] = *l* such that TXT[SA[*l*]:] ⩽ *q* < TXT[SA[*l*] + 1:]. SA and SA_Q_ are combined using the merge-sort algorithm knowing that SA and SA_Q_ are already sorted which results in a linear-time algorithm.

### 3.2 Sequence comparison

SANS and KSEARCH use simple scores to sort the list of database proteins and return the top H hits or all hits with a score above a threshold T. SANS estimates similarity using a SANS, introduced here [Equation (1)]. KSEARCH estimates similarity based on *k*-mer composition [Equation (3)].

The SANS gives a positive score to those database proteins (sprot), which have the longest common prefixes with a query suffix. The score is accumulated over all suffixes of the query protein (qprot) as follows:
(1)
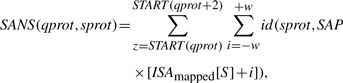

where the width of the window *W* is a parameter and the identity function *id* (*a,b*) is
(2)
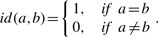

The top *H* matches with the highest SANS scores are output. We set *W* = *H* by default. Whether the best matching suffixes are lexically smaller or greater than the query suffix, at least *H* such matches will be included in the window.

KSEARCH score is the dot product of the feature vectors F:
(3)
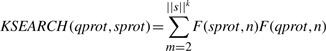

where the feature vectors F(x,n) say how many times a given *k*-mer *n* occurs in the protein *x*. |S| is the size of the alphabet. We note that the use of suffix arrays allows the user to freely set the word length parameter *k*.

Short tandem repeats can give rise to artificially high scores. We mask query suffixes which have the same character in positions (1,2,3) or positions (1,3,5). Masked suffixes are ignored and do not contribute to the SANS score or KSEARCH score.

### 3.3 Complexity analysis

Sais-lite is a light-weight suffix array construction algorithm that takes linear time and space (Nong *et al.*, 2009). The merge-sort step is also linear O(|TXT|+|TXT_Q_|). Treating the width of the window as a constant, the search step of SANS does O(|TXT_Q_|) lookups in the suffix array SA. The overall time complexity of the SANS algorithm is thus O(|TXT|+|TXT_Q_|). The KSEARCH algorithm checks all instances of *k*-mers in the database, and the number of these instances grows proportionally to the size of the database. The overall time complexity of the KSEARCH algorithm is thus O(|TXT| * |TXT_Q_|).

### 3.4 Greedy approximate alignment

Sequence alignment is slow. We implemented a greedy procedure which processed a few million pairwise alignments per hour. The procedure is faster for highly similar sequences. It starts similarly to BLAST looking for diagonals with two hits of closely spaced 3-mers (at distances up to 40 amino acids). If a two hit is found, the algorithm tries to extend the alignment without gaps, resulting in a high-scoring segment pair (HSP). HSPs are scored using BLOSUM62 substitution scores. The query sequence is scanned for two hits from beginning to end. If an HSP is detected, the scan jumps to the end of the HSP. In other words, potential 3-mer seeds already within an HSP are not tested. When the end of the query sequence has been reached, all HSPs are sorted based on their scores. A compatible set is selected so that the HSPs form a sequential alignment. Gap penalties are not used. Greedy alignments can be generated optionally for the hits detected by the SANS or KSEARCH algorithms.

## 4 IMPLEMENTATION

Suffix arrays were computed using the recursive SAIS algorithm (Nong, 2009). The in-house programs were written in Fortran-95 and compiled using gfortran. Evaluations were done using Perl scripts. All computations were done on Linux computers with 64 or 500 Gb RAM.

## 5 RESULTS AND DISCUSSION

Traditionally, word filter heuristics are used to speed up database searches. The suffix array gives access to seed words of arbitrary length. We performed a comprehensive set of benchmark tests to evaluate both fixed size patterns and suffix array neighborhoods.

### 5.1 Metagenome benchmark

The metagenome benchmark is chosen to represent new sequences not yet present in the database. When a mapping exists, the median identity between the metagenomic and the closest database proteins is ~50%. This is suitably challenging, as detecting exact matches is trivial and exact filters can be formulated for very high levels, i.e. > 90% of sequence identity (Holm and Sander, 1997).

**Fig. 4.**
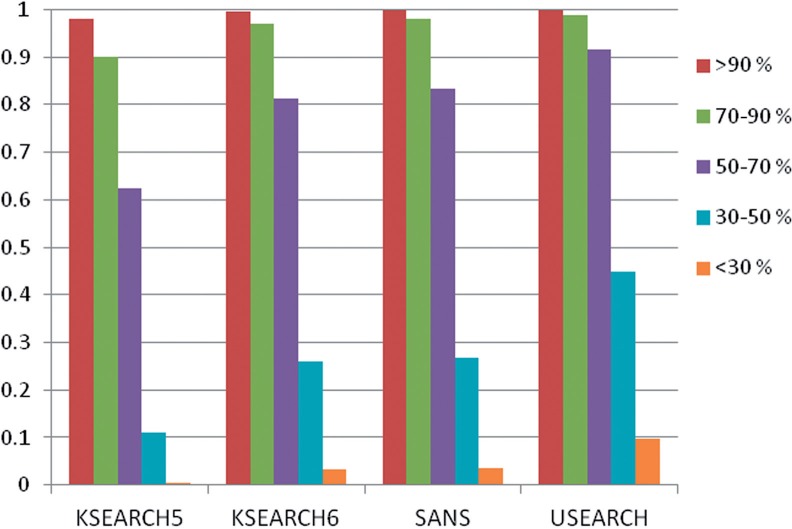
Correctness of mappings in metagenome benchmark. The top hit of 3.6 million queries were evaluated using BLAST (*e*-value < 1) as reference of truth. USEARCH was run with default parameters (*a* = 1, *r* = 8). SANS was run with window size 1. KSEARCHx were run with word size *x*

We compared SANS and KSEARCH to USEARCH on the metagenome versus swissprot benchmark (Fig. 4). The 32-bit version of USEARCH, which is available academically, ran out of memory on larger databases than swissprot (a 64-bit version is available commercially). We evaluate a mapping as successful if the top hit is found in the BLAST list for the query. KSEARCH shows the worst performance. The performance of SANS is close to the performance of USEARCH although SANS is 10 times faster and USEARCH has the advantage of multiple testing. USEARCH generates explicit alignments of the top hits until it finds an ‘accept’ with *e*-value <1 or eight ‘rejects’ (Edgar, 2010).

SANS generated top-hit mappings of the metagenome set against swissprot in 2 h compared to 20 h by USEARCH (Table 4). SANS also scales well to the larger Uniprot database. We note that the time taken to generate greedy alignments actually went down. This is because there are more close hits to be found in Uniprot which are quicker to align.

### 5.2 Genome benchmark

SSEARCH was used to generate a reference of truth for the genome benchmark, enabling the evaluation of selectivity and sensitivity in different ways. Precision and recall are used synonymously with selectivity and sensitivity.

**Fig. 5.**
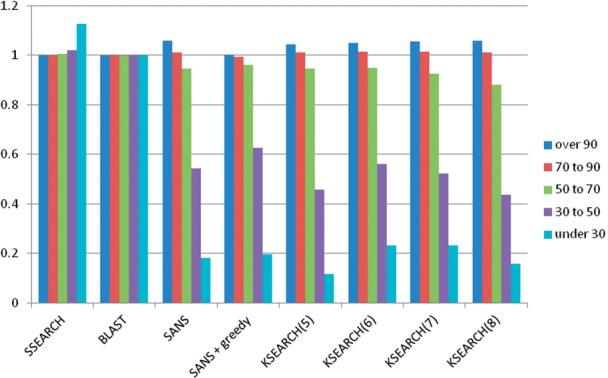
Relative sensitivity of SSEARCH, BLAST, SANS and KSEARCH on the genome benchmark. The top-1000 hits per query are evaluated. Sensitivity is calculated for different bins of sequence identity. Sensitivities within each bin are scaled linearly so that BLAST sensitivity is one. Sequence identities of the TP hits were taken from SSEARCH. SSEARCH and BLAST hits are sorted by *e*-value. SANS was run with *W* = *H* = 1000. SANS+greedy was run with *W* = *H* = 2000 and the top-2000 hits were reranked by greedy alignment score, keeping the top-1000. KSEARCH(k) were run with word length *k*

Word filters are as sensitive as BLAST in the 50–100% regime of sequence identity (Fig. 5). The best method (SANS+greedy) has relative sensitivities of 96–100% in this regime. *k* = 6 consistently gives the optimum performance for KSEARCH. The largest differences between methods emerge in the 30–50% regime. We tried a number of variations but only reached ~70% sensitivity relative to BLAST in the 30–50% regime. The tested variations included (i) rescoring hits according to an alignment score, (ii) varying the width of the window in SANS, (iii) hybrids combining the SANS and KSEARCH scores (sum performed best), (iv) a two-hit filter like that in BLAST and (v) distance-dependent id function (cf. Equation 2). The best method (SANS+greedy) took the top-2000 hits by SANS and rescored the hits by greedy alignment. In the 30–50% identity range this improved sensitivity to 63%, up 9% points from SANS. Rescoring KSEARCH hits generally brought but little improvements. In the low sequence identity range (<30–50%), all methods except BLAST miss a large fraction of true hits.

**Fig. 6.**
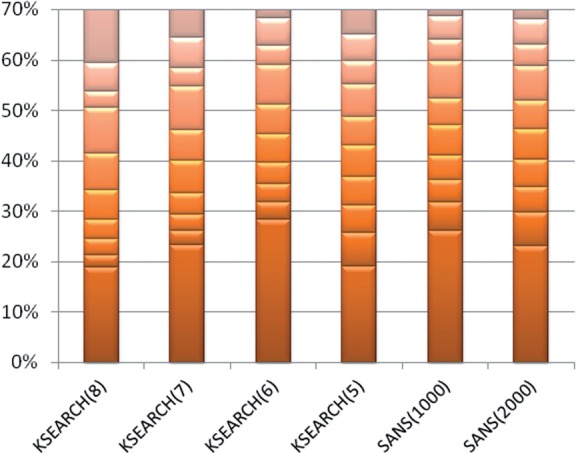
Distribution of AUC values from 4173 query-specific precision-recall plots of the genome benchmark. The top-1000 hits per query were evaluated. AUC values are binned into 10 evenly spaced intervals between one (bottom, perfect result) and zero (gray at top, total failure). KSEARCH runs are labeled with word size (*k*). SANS runs are labeled with the window size (*w*). SANS(1000) has the highest average

In terms of a precision–recall plot, Figure 5 tests the sensitivity at 1000 ‘positives’. Figure 6 compares the SANS and KSEARCH scores in terms of the area under the curve (AUC) until 1000 positives. Higher AUC corresponds to better internal ranking of the true positives above false positive hits. All methods have large peaks at AUC values near zero (bad) and near one (good). The overall average of SANS is the highest (Table 3). KSEARCH performance peaks at *k* = 6 and greedy alignment rescoring always improves performance (Table 3).

**Table 3.** Average AUC values per query in genome benchmark

Score for sorting	KSEARCH				SANS		BLAST
	*k* = 5	*k* = 6	*k* = 7	*k* = 8	*w* = 1000	*w* = 2000	
Native	0.51	0.55	0.49	0.44	0.56	0.54	
Alignment	0.52	0.56	0.49	0.44	0.60	0.62	0.95

**Table 4.** Comparison of running times

Query set	Database	Program	Parameters	Time		
				Indexing^a^	Search	Alignment
Uniprot	uniprot	SANS	*W* = 100	3 h 22 m	30 h 4 m	2 h 19 m
Metagenome	swissprot	BLAST	–b 250		380 d	
		USEARCH	*a* = 1 *r* = 8		19 h 55 m	
		SANS	*W* = 1	57 m	13 m	48 m
	uniprot	SANS	*W* = 1	4 h 52 m	28 m	32 m
Genome	uniprot	SSEARCH	–s BL62 –f −11 –g −1 –E 1.0 –m 9C –z 3 –d 0		640 d	
		BLAST	–b 1000		100 h	
		parallel BLAST	10 processors, –b 1000		max 13 h / processor	
		SANS	*W* = 100	3 h 57 m	12 m	10 m
			*W* = 1000		23 m	52 m
			*W* = 2000		33 m	1 h 40 m

^a^Indexing time includes indexing both the query set and database from scratch.

### 5.3 Speed

SANS searches scale independent of database size. The search time scales roughly O(*WN*) where *W* is the width of the suffix array neighborhood and *N* is size of the query set. Alignment is the rate-limiting step of many fast filters. The SANS score can be used for ranking hits without alignment. These advantageous properties make SANS the fastest word filter that we know. Concretely (Table 3), SANS was 10 times faster than USEARCH in the metagenome benchmark. The genome benchmark showed that scanning a new genome against uniprot takes only 1 h. This is 100 times faster than BLAST though it must be said that, in practice, BLAST is often run in parallel.

### 5.4 Memory

The proliferation of suffix tree/suffix array applications has been hampered by their excessive memory usage in the past. Our implementation of SANS is light-weight in terms of memory. This is achieved by computing the suffix arrays separately for sections of the database and by searching the database in sections so that only a section of the indices need to be loaded in memory at one time. The amount of memory used by the program can be set by the user (MEMORY parameter). The minimum requirement is one byte per amino acid. The current Uniprot database is ~8 Gb. The program runs comfortably on Uniprot with MEMORY set to 10–16 Gb. The ISA_mapped_ and SAP indices used by the search step occupy 8m+4n bytes of disk space, where *n* is the total length of database sequences and *m* is the total length of query sequences.

## 5.5 Conclusions

We have investigated the use of word filters to speed up protein sequence database searches. The principal conclusions from our extensive benchmarking can be summarized as follows:

1. word filters are as sensitive as BLAST in the *feasible regime* of 50–100% sequence identity,
2. many variants of word filters perform about equally well in the feasible regime but SANS is the most robust to parameter variation,
3. suffix array supports the fastest known word filter algorithm,
4. methods incorporating explicit alignment are necessary *<*50% identity.

We introduced the SANS which has attractive scaling properties. It processes millions of queries in a matter of hours and scales to large protein sequence databases with billions of amino acids. It supports alignment-free mapping and is 10 times faster than the fastest known program for protein mapping. SANS makes the database comparison problem local which gives it good speed. In comparison to traditional word filters, we found that SANS behaves more robustly with respect to parameter variation. For example, varying window width had little effect on SANS, whereas varying word size *k* had a large effect on KSEARCH (Fig. 5). The complexity of the SANS algorithm is O(n+m). The program is the more efficient, the larger the batch of query sequences is. For single queries, BLAST remains the best option.

BLAST is a widely used, general purpose homology search tool. SANS has more restricted use. It is designed for the comparison of protein sequences and it only reports a user-specified number of nearest neighbors. It provides useful input to any kNN classifier, including applications in EST mapping, ortholog mapping, protein function assignment and family membership assignment.

We observed that word filters get very close to the sensitivity of BLAST when sequence identity is 50–100%. Similar observations have been made previously on simulated data and domain segments (Edgar, 2010). The feasible regime of protein sequence retrieval by word filters coincides with the regime of reliable function transfer. Minimum sequence identities above the 40% to 70% level have been recommended by various researchers (Devos and Valencia, 2000; Friedberg, 2006; Lee *et al.*, 2006; Rost, 2002).

Modern function annotation tools are designed to make an optimal choice between conflicting candidates for function assignment. They take a ranked list of sequence neighbors as input, group neighbors with similar functional descriptions and calculate an aggregate weighted support for each candidate prediction outcome (Kankainen *et al.*, 2012; Koskinen *et al.*, unpublished). Assuming that false positives hits generate a low random background, it may be possible to use SANS in its fastest, alignment-free mode to generate hit lists for assigning functions to new genomes.

Our benchmark gives a realistic picture of the challenges in metagenome or genome research. The following calculation suggests that putting a word filter in front of BLAST to eliminate ‘easy’ queries would significantly reduce the work load in genome annotation. One BLAST run against Uniprot takes 1 or 2 min. The default number of hits returned by BLAST is 250. In our bacterial genome benchmark, 53% of the queries produce at least 250 hits (ordered by *e*-value) which are all *>*50% identical to the query. The genome benchmark was purposely selected from a phylum with relatively few known sequences. There are phyla with hundreds of closely related strains and species sequenced, and in these cases the word filter could do all the work.
